# Following Up Patients With Chronic Pain Using a Mobile App With a Support Center: Unicenter Prospective Study

**DOI:** 10.2196/60160

**Published:** 2025-01-22

**Authors:** Marta Antonia Gómez-González, Nicolas Cordero Tous, Javier De la Cruz Sabido, Carlos Sánchez Corral, Beatriz Lechuga Carrasco, Marta López-Vicente, Gonzalo Olivares Granados

**Affiliations:** 1 Department of Neurosurgery, Hospital Universitario Virgen de las Nieves, Av. Juan Pablo II s/n, Granada, 18013, Spain, 34 699699250; 2 Department of Human Anatomy, University of Granada, Granada, Spain

**Keywords:** pain management, mobile health, mHealth, eHealth, chronic pain, support center, mobile phone app, survey, follow-up, pain control, prospective study

## Abstract

**Background:**

Chronic pain is among the most common conditions worldwide and requires a multidisciplinary treatment approach. Spinal cord stimulation is a possible treatment option for pain management; however, patients undergoing this intervention require close follow-up, which is not always feasible. eHealth apps offer opportunities for improved patient follow-up, although adherence to these apps tends to decrease over time, with rates dropping to approximately 60%. To improve adherence to remote follow-up, we developed a remote follow-up system consisting of a mobile app for patients, a website for health care professionals, and a remote support center.

**Objective:**

Our objective was to evaluate patient adherence to remote follow-up using a system that includes a mobile app and a remote support center.

**Methods:**

After review of the literature and approval of the design of the follow-up system by a multidisciplinary committee, a team of experts developed a system based on a mobile app, a website for health care professionals, and a remote support center. The system was developed in collaboration with health care professionals and uses validated scales to capture patients’ clinical data at each stage of treatment (ie, pretreatment phase, trial phase, and implantation phase). Data were collected prospectively between January 2020 to August 2023, including the number of total surveys sent, surveys completed, SMS text message reminders sent, and reminder calls made.

**Results:**

A total of 64 patients were included (n=40 women, 62.5%) in the study. By the end of the study, 19 (29.7%) patients remained in the pretreatment phase, 8 (12.5%) patients had completed the trial phase, and 37 (57.8%) reached the implantation phase. The mean follow-up period was 15.30 (SD 9.43) months. A total of 1574 surveys were sent, along with 488 SMS text message reminders and 53 reminder calls. The mean adherence rate decreased from 94.53% (SD 20.63%) during the pretreatment phase to 65.68% (SD 23.49%) in the implantation phase, with an overall mean adherence rate of 87.37% (SD 15.37%) for the app. ANOVA showed that adherence was significantly higher in the earlier phases of treatment (*P*<.001).

**Conclusions:**

Our remote follow-up system, supported by a remote support center improves adherence to follow-up in later phases of treatment, although adherence decreased over time. Further studies are needed to investigate the relationship between adherence to the app and pain management.

## Introduction

Chronic pain is one of the most common conditions globally and is associated with reduced quality of life, increased medical expenses, and significant economic costs [1]. Its prevalence ranges between 2%‐40% [1-3], and annual health care costs due to pain amount to US $300 billion in the United States [1] and €12 billion ( US $12.3 billion) in Norway [2].

The treatment of chronic pain requires a multidisciplinary approach, with spinal cord stimulation being a potential treatment option. This technique is safe and effective [4] and has also shown benefits in management of other chronic pain conditions, such as complex regional pain syndromes, low back pain, and other forms of pain. However, ensuring consistent follow-up for these patients is challenging, as they require close monitoring, which is not always feasible.

New technologies have opened up a lot of possibilities to address this challenge. The World Health Organization defines eHealth as the cost-effective and secure use of information and communication technologies to support health and health-related areas, including health services, health monitoring, health literature, health education, and knowledge and research [5].

There are numerous tools designed to facilitate medical care, including mobile apps, websites, and other platforms. While there is limited evidence to support their use, preliminary results are promising. For example, mobile apps used alongside conventional treatments have shown better results in the management of chronic pain than conventional interventions alone [6,7].

eHealth apps also offer an opportunity for closer patient follow-up, although adherence to treatment tends to decline over time, dropping to approximately 60%, as previous studies have shown [8,9]. To address this challenge, we have developed a system that includes a mobile app for patients, a website for health care professionals, and a remote support center.

The aim of this study is to assess patient adherence to follow-up care using a mobile app supported by a remote support center.

## Methods

### Overview

In 2019, researchers at the Department of Pain Surgery at Hospital Virgen de las Nieves in Granada, Spain, initiated approaches to improve the remote monitoring of patients implanted with epidural spinal cord stimulators for chronic pain management. The aim was to reduce the number of face-to-face consultations or hospital visits while providing additional support to these patients. Therefore, we decided to develop an app complemented by a support center to improve patient monitoring as much as possible. After reviewing the relevant literature, we adapted the tool kit published by Marvel et al [10] to suit our needs, following the four key steps outlined in Table 1. As we already had a multidisciplinary group, we sought the agreement of existing team members and assembled a group of experts. Instead of accelerating the project, we adapted our current workflows and protocols to the new working framework. We also developed the follow-up system after adapting the protocols already in place and enrolled patients only after the system was finalized.

**Table 1.** Project phases.

Johns Hopkins tool steps^a^	Project workflow (current study)
Define the problem and the digital tool	Define the problem and the digital tool
Creation of a multidisciplinary group	Creation of an expert team, approval by a multidisciplinary group
Seeking opportunities to accelerate the project	Adaptation of the existing clinical and educational protocol into the framework of the remote follow-up system
Involving professionals	Designing the follow-up system, considering the needs of the involved professionals, and subsequent enrollment of patients
Consulting different partners	Performing quality assessment
Conducting a clinical validation	Conducting a clinical validation

a Marvel et al [10].

The concept of a remote follow-up system was presented to a multidisciplinary committee composed of anesthesiologists, neurologists, neurosurgeons, neurophysiologists, and rehabilitation specialists. This committee meets monthly to assess patients with chronic pain who may benefit from interventional therapies such as spinal cord stimulation when conservative treatments have failed. The target population was identified as patients who could benefit from spinal cord stimulation and had not yet started the trial phase, to enable data collection before and after implantation.

The inclusion criteria for patients eligible for spinal cord stimulation system implantation at our center are shown in Textbox 1.

Textbox 1. **Inclusion criteria.**

- Chronic pain in a specific area of the body (patients experiencing diffuse pain were not accepted)
- Failure of conservative treatments, including rehabilitation and local infiltration
- A favorable psychological evaluation confirming that the patient can wear a spinal cord stimulation system
- No contraindications to surgery or allergies to system components
- No history or current substance use, especially opioids

**Figure 1.**
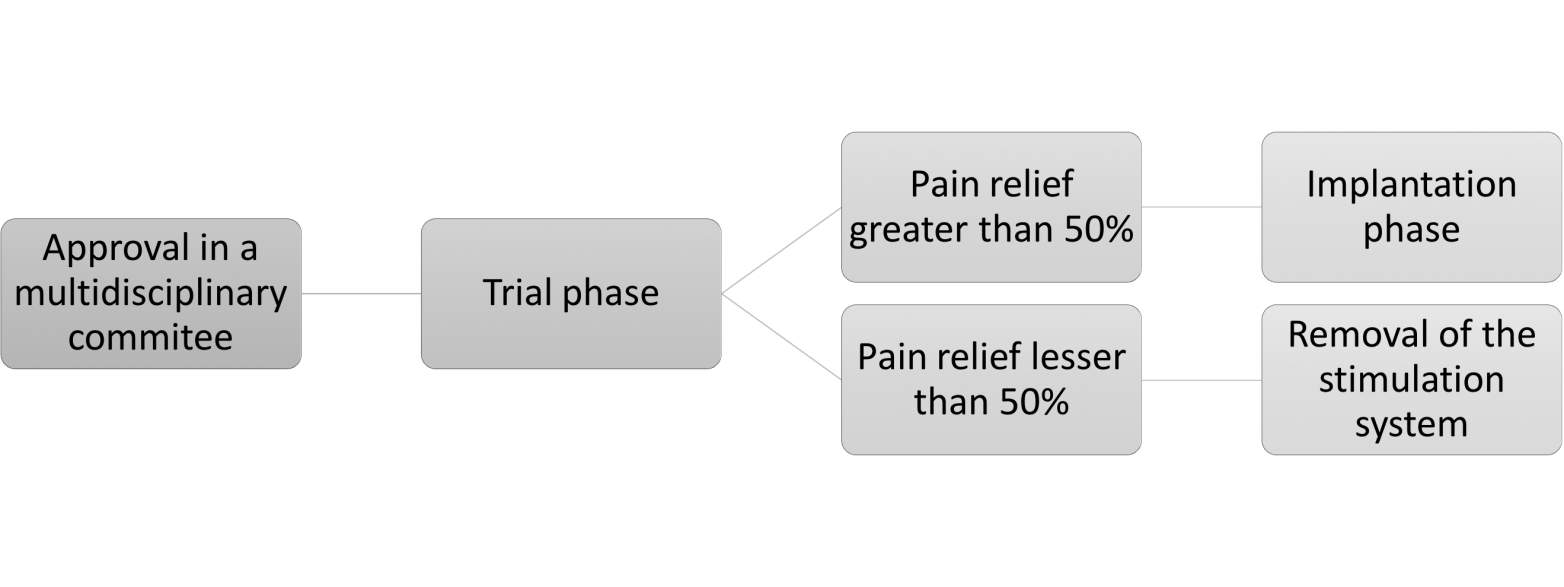
Algorithm used to evaluate the implantation of the spinal cord stimulation system.

Once the committee had approved the implantation of a spinal cord stimulator, the procedure was performed in 2 phases, as shown in Figure 1. In the trial phase, one or more electrodes were implanted in the epidural space and connected to an external stimulator. The efficacy of the system was then assessed over a 4-week period using the verbal numerical rating score (VNRS). The intervention was considered effective if the patient’s reported subjective perception of pain relief was at least 50%. If the trial phase was successful, it was followed by the implantation phase, in which a permanent stimulator was implanted in the adipose tissue of the abdomen or lower back. If the 50% threshold was not reached, the system was removed completely.

### Ethical Considerations

Ethics approval was obtained from the Comité de Investigación Provincial de Granada (study: NC-D-01; ethics committee reference: SICEIA-2020‐000438), and informed consent for the procedure and follow-up system was obtained from all included patients. To maintain privacy, each patient was assigned a code with a number so that only health care professionals could access the personal data. The platform meets all the standards of Regulation (EU) 2016/679 of the European Parliament and of the Council of April 27, 2016, and has been approved by local and state authorities. This ensures that only health care professionals can access all the data collected and that data security is contractually guaranteed. The study participants have not been compensated in any way.

### Design of the Follow-Up System

The remote follow-up system consists of 3 main components: a mobile app for patients, a website for health care professionals, and a remote follow-up center. The mobile app was designed to ensure close monitoring during all phases of treatment, using feedback from all health care professionals involved. The app was developed in collaboration with Clinical Care Connect (Persei) and customized by our team to meet our specific needs. It offers various features including educational content such as brochures and videos, questionnaires with validated scales, pop-up notifications, and a form to contact the remote support center. The validated scales used include Doleur Neuropathique 4 (DN4) to assess neuropathic pain, Oswestry Disability Index/Neck Disability Index (ODI/NDI) to assess back pain, VNRS to assess pain, and 36-item Short Form Survey (SF-36) to assess quality of life. In addition, patient-reported experience measures were used to assess experience and satisfaction with the app. Since pain is a subjective perception, the use of validated scales can help to measure it and compare it between patients.

The website designed by Persei (Vivarium S.L., Spain) can be accessed from anywhere with the necessary authorizations. It also provides alerts using different colors depending on the urgency of the detected problem: red for infection, yellow for insufficient pain relief, and blue for low battery. These alerts are monitored by the remote support center, which contacts the specialist by making a telephone call if necessary, such as, in the event of an infection. For nonemergency issues, such as insufficient pain relief, the support center contacts the patient to analyze the cause, and a priority appointment for treatment is scheduled. This remote support center was set up to increase the efficiency of treatment and improve pain management. The aim was to maximize treatment adherence and ensure the sustainability of the remote follow-up system. Previously, follow-up was carried out 1 month after implantation, 1 year after implantation, and subsequently, as required.

### Data Collection and Analysis

We collected data from all patients who were approved for spinal cord stimulation between January 2020 and August 2023 and enrolled in the remote monitoring program. If the committee approved the patient for spinal cord stimulation, informed consent for the remote follow-up system was obtained in addition to consent for implantation. Data were collected automatically and prospectively through surveys completed via the app. The questionnaires were distributed according to the protocol shown in Textbox 2.

Textbox 2. **Protocol used to send validated scales.**

**Validated scales used in the protocol used by the Virgen de las Nieves Hospital**

- Pretreatment phase: Doleur Neuropathique 4 (DN4), verbal numerical rating score (VNRS), Oswestry Disability Index/Neck Disability Index (ODI/NDI), 36-item Short Form Survey (SF-36)
- Trial phase: Daily VNRS and patient-reported experience measures (PREMs) at the end of the trial phase
- Implantation phase: DN4, VNRS, ODI, and SF-36 after 1 and every 3 months; the PREMs survey was also distributed after 12 months

The support center sends a notification each time a survey needs to be completed, for a maximum of 3 times. If the questionnaires were not completed, a notification was sent to the hospital so that a specialized caregiver can contact the patient. One of the biggest challenges was ensuring privacy. To achieve this, each patient was coded with a number so that only health care professionals had access to the personal data and that data security was contractually guaranteed.

The data were collected automatically when a survey was answered via the app and stored in a private cloud. This allowed us to minimize potential errors. In addition, the support center guaranteed that the data collected via the app belonged to our hospital’s patients and was only accessed and analyzed when a specialist at our center requested it. Demographic information was collected for data analysis. Adherence was measured by calculating the percentage of completed surveys relative to the total number of surveys sent. We analyzed adherence rate during each treatment phase and for the overall study, and compared adherence by demographic variables using ANOVA, adjusting for gender, age, diagnosis, and phase. Age was categorized into 2 groups, with 40 years as the cutoff point, since previous studies have found significant differences in prevalence rates for chronic pain and quality of life [11]. Regarding completeness, we only considered a survey as answered if it was completed. If data were missing, a notification was sent to the patient to complete the survey.

## Results

Between January 2020 and August 2023, a total of 64 patients were enrolled in the study, comprising 24 (37.5%) men and 40 (62.5%) women. While identifying the cause of pain, 32 (50%) patients had complex regional pain syndromes, 18 (28%) had failed back syndrome, and 14 (22%) had other diagnoses such as phantom limb syndrome, adhesive arachnoiditis, coccydynia, and axonotmesis. Before the onset of pain, 19 (28%) patients had no fixed occupation, while 10 (18%) were involved in nonphysical and 35 (55%) in physical occupations such as farming or construction (Table 2).

**Table 2.** Summary of epidemiological data.

Variables	Patients (N=64), n (%)
Gender	
Men	24 (38)
Women	40 (63)
Age interval	
<40 years	9 (14)
≥40 years	55 (86)
Diagnoses	
CRPS^a^	32 (50)
FBS^b^	18 (22)
Others	14 (18)
Occupation	
Physical occupation	35 (55)
Nonphysical occupation	19 (18)
No fixed occupation	10 (28)
Phase	
Pretreatment	19 (30)
Trial	8 (13)
Implantation	37 (58)

a CRPS: chronic regional pain syndrome.

b FBS: failed back syndrome.

At the end of the study, 19 (30%) patients were in the pretreatment group, awaiting the start of the trial phase. A total of 8 (13%) patients reached the trial phase but were not transferred to the implantation phase due to insufficient improvement in pain management. Further, a total of 37 (58%) patients advanced to the implantation phase (Table 2).

**Figure 2.**
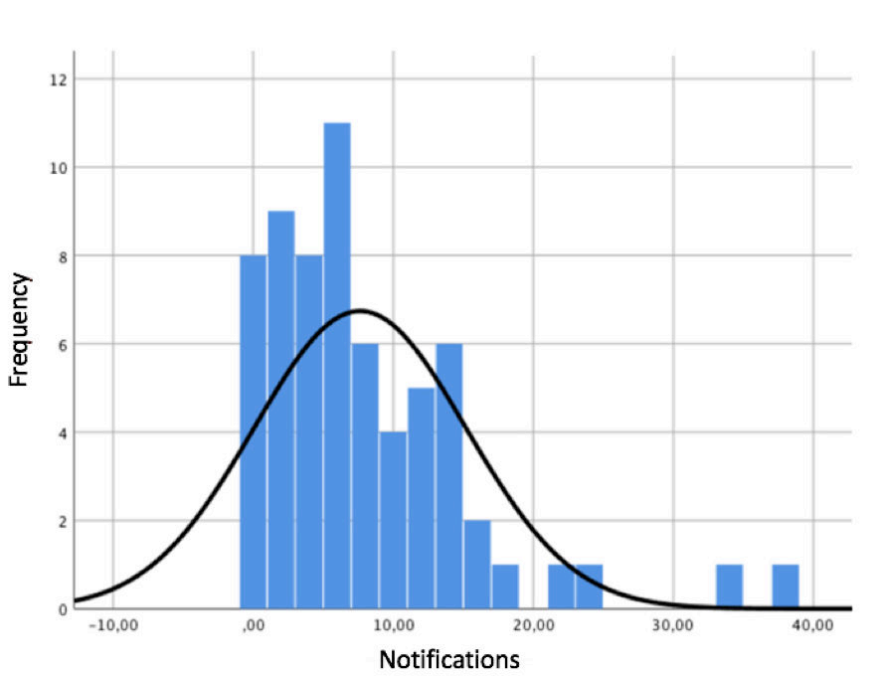
Histogram with a normality curve showing the number of patients in groups (frequency) based on the number of required notifications (notifications).

A total of 1586 surveys were distributed during the study. SMS text message notifications had to be sent to the patients 488 times, which corresponded to a mean of 7.62 (SD 7.57) SMS text messages per patient. However, 8 (13%) patients did not require SMS text messages notifications, while the remaining (n=56, 88%) patients required at least one notification (Figure 2). It was observed that several patients required more than 30 notifications. In addition, a total of 53 phone calls were made to remind patients to complete the surveys; however, 43 (67%) patients did not require any reminder calls.

The mean follow-up time was 15.38 (SD 9.43) months. A total of 12 (19%) patients were lost to follow-up, and 3 (4%) patients discontinued using the app (ie, 1 during the pretreatment phase and 2 patients during the implantation phase). For patients who discontinued using the app, any surveys not answered by them were considered failures.

**Figure 3.**
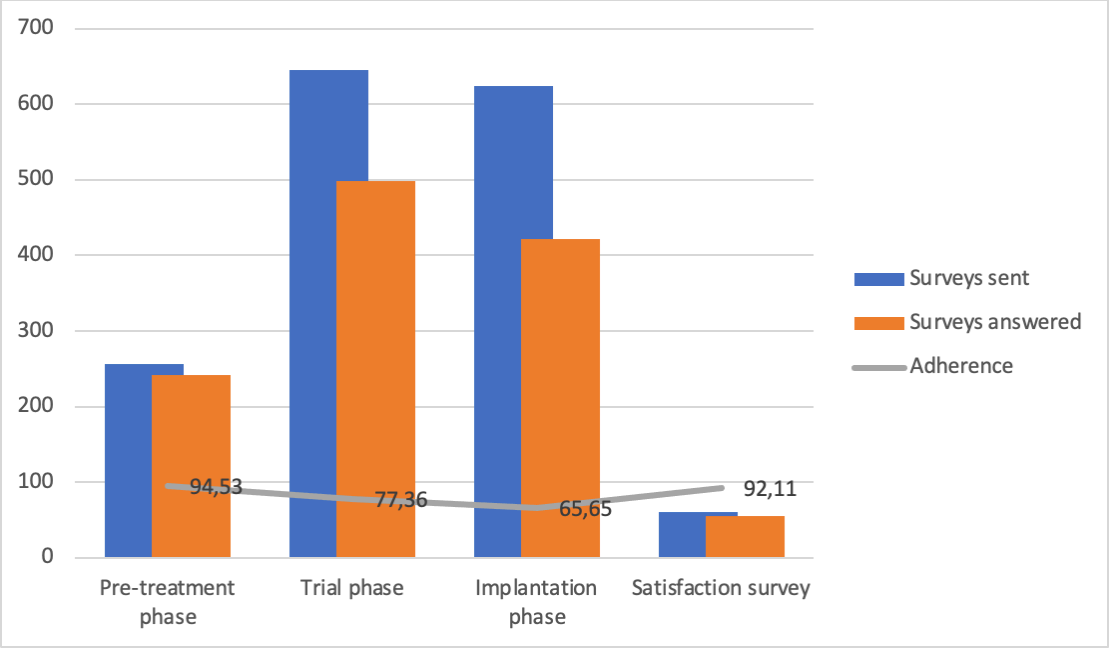
Surveys and adherence rate.

Adherence was analyzed for each phase and overall, as shown in Figure 3, which represents the total number of surveys sent and answered in each phase, as well as the adherence rate in each phase. During the pretreatment phase, 256 surveys were sent, of which 242 were completed, corresponding to a mean adherence rate of 94.53% (SD 20.63%). During the trial phase, 645 surveys were sent, of which 499 were answered, corresponding to a mean adherence rate of 77.36% (SD 37.01%). During the implantation phase, 624 surveys were sent, of which 422 were answered, corresponding to a mean adherence of 65.68% (SD 23.49%). A total of 61 satisfaction surveys were sent (excluding 3 patients who discontinued using the app), of which 55 were answered, corresponding to a mean adherence rate of 92.11% (SD 18.47%). The overall mean adherence rate for using the app was 87.37% (SD 15.37%) across all phases (Table 3).

**Table 3.** Summary of surveys sent and answered and adherence in each phase.

Phase	Surveys sent, n^a^	Surveys answered, n^a^	Adherence rate^b^ (%), mean (SD^c^)
Pretreatment phase	256	242	94.53 (20.63)
Trial phase	645	499	77.36 (37.01)
Implantation phase	624	422	65.68 (23.49)
Satisfaction survey	61	55	92.11 (18.47)
Total adherence	1586	1218	87.37 (15.37)

a Number of surveys during each phase.

b Adherence rate was defined as the total surveys answered x 100/total surveys sent.

c The SD represents the variability of adherence rates among individual patients.

ANOVA revealed that adherence rates were significantly higher in the earlier phases of treatment (*P*<.001). There was also a trend indicating that women were more likely to complete satisfaction surveys (*P*=.17) and surveys in general (*P*=.64). However, women also required more SMS text message reminders (*P*=.70) and phone calls (*P*=.71). No significant differences in treatment adherence were found when comparing younger patients versus patients older than 40 years or when comparing adherence to treatment based on the cause of pain or type of occupation (Table 4).

**Table 4.** Summary of the ANOVA results.

Variables	Pretreatment phase adherence (%; n=64), mean (SD)	*P* value	Trial phase adherence (%; n=43), mean (SD)	*P* value	Implantation phase adherence (%; n=38), mean (SD)	*P* value	Satisfaction survey adherence (%; n=38), mean (SD)	*P* value	Global adherence (%), mean (SD)	*P* value
Gender		.81		.49		.18		.40		.80
Total	94.5 (20.6)		66.7 (23.5)		65.7 (23.5)		92.1 (18.5)		87.4 (15.4)	
Men	93.7 (22.4)		58.2 (31.3)		58.2 (31.3)		95.8 (14.4)		86.8 (19.1)	
Women	95.0 (19.8)		69.1 (18.6)		69.1 (18.6)		90.4 (20.1)		87.7 (12.9)	
Age (years)		.38		.29		.74		.90		.30
Total	94.5 (20.6)		77.36 (37.0)		65.7 (23.5)		92.1 (18.5)		83.4 (15.4)	
≤40	88.9 (33.3)		62.9 (42.2)		68.4 (19.9)		92.9 (18.9)		82.5 (14.9)	
>40	95.5 (18.1)		80.2 (34.6)		65.1 (25.6)		91.3 (18.7)		88.2 (15.4)	
Diagnoses		.42		.63		.57		.83		.35
Total	94.5 (10.6)		77.4 (37.0)		65.7 (23.5)		92.1 (18.5)		87.4 (15.6)	
CRPS^a^	100 (0)		72.8 (42.2)		63.1 (29.9)		92.8 (17.9)		85.7 (16.9)	
FBS^b^	94.5 (21.8)		81.7 (30.2)		73.5 (12.8)		93.8 (17.7)		92.7 (9.7)	
Others	90.3 (25.9)		85.3 (28.3)		64.8 (9.7)		88.9 (22.0)		86.2 (15.9)	
Phase		.61		.51		.60		.67		.001
Total	94.5 (20.6)	—^c^	—	—	87.4 (15.4)
Pretreatment	96.1 (12.5)		77.8 (21.1)		77.8 (0)		100 (0)		96.1 (12.5)	
Trial	100 (0)		65.4 (4)		65.4 (23.8)		91.9 (18.7)		94.3 (9.2)	
Implantation	92.6 (25.6)		77.4 (37.0)		65.7 (23.5)		92.1 (18.5)		81.4 (15.2)	

a CRPS: complex regional pain syndromes.

b FBS: fail back syndrome.

c Not applicable.

## Discussion

### Comparison With Previous Research

In recent years, there has been increasing interest in the use of pain control apps for various purposes, such as postsurgical follow-up, pain self-management, and chronic pain management [6-10,12-14]. However, many of these tools have not been as effective as expected, often due to inadequate planning and a lack of preliminary studies to anticipate necessary roles [15]. However, some studies have demonstrated that these apps can outperform traditional follow-up methods [6,14]. Our study introduces a remote support center, a feature that we believe is not yet widely adopted internationally. The aim of this study is to determine whether the inclusion of a support center improves adherence compared to other systems.

An analysis of the medical apps across various app stores shows that approximately 86% of these apps were developed without the involvement of medical professionals [16]. Lalloo et al [17] examined 1019 apps developed for postoperative pain management or education and found that only 10 apps met the established criteria. When evaluating apps developed with the involvement of health care professionals, only 5 (0.49%) of them were deemed suitable. Similarly, Bhattarai et al [16] examined 373 apps focused on arthritis pain self-management with only 4 apps meeting the Stanford criteria for pain self-management. Portelli and Eldred [18] evaluated 195 apps for pain management against the guidelines for cognitive behavioral therapy. Of these, only 6 (3%) of these apps met the standards, leading to the conclusion that neither health care professionals nor patients were involved in their development. Despite these limitations, a recent meta-analysis of 4767 patients in 22 randomized trials found that these apps offer a small but significant improvement in long-term pain management [19].

For patients undergoing spinal cord stimulation, close follow-up is required to adjust the type and parameters of stimulation, which usually necessitates face-to-face visits. This increases the time and cost burdens for patients and caregivers, especially for those who live far from specialized centers [20]. Our remote follow-up system was developed to overcome these challenges and ensure optimal patient monitoring. The mobile app in combination with the remote support center reduces the need for in-person examinations while ensuring a high standard of care. The app’s alert system helps to detect problems at an early stage, such as infections or low battery levels so that immediate action can be taken.

In addition, the use of validated scales during an in-person consultation is challenging due to the limited time available. Consequently, this system has allowed a better understanding of the patient’s condition. Furthermore, some patients have found that expressing themselves via the app helps them [20] in experiencing privacy and without any time pressure.

Adherence to medical app use is crucial as studies show that 75% of users stop using an app within 48 hours of downloading, and 15% delete it after initial use [21]. A review by Wikström et al [9] found that none of the studies focused on improving adherence or motivating users to continue using medical apps. The easiest way to increase motivation is through notifications, which is why our study focused on the role of the remote support center. We consider this as a key factor in the success of our study. However, numerous motivational elements in medical apps have been analyzed in previous studies, as summarized in Table 5.

**Table 5.** Motivational elements in eHealth apps.

Studies	Motivational elements								
	Tablet lent to the patient	Chat	Involvement of relatives	Educational texts, pictures, and/or videos	Follow-up of auto evaluation and graphic results	Personalized follow-up	Sharing media elements	Notifications and reminders	Alerts
Alam et al [20]		✓^a^		✓				✓	
Pecorelli et al [22]	✓			✓	✓			✓	
Perdoncini et al [23]		✓		✓					
Shah et al [24]		✓		✓					
Davidovitch et al [25]		✓		✓			✓		
Felbaum et al [26]		✓		✓			✓	✓	
Glauser et al [27]		✓		✓	✓		✓	✓	
Gustavell et al [28]		✓		✓	✓			✓	✓
Hou et al [29]		✓		✓		✓		✓	✓
Pickens et al [30]		✓	✓	✓				✓	
Timmers et al [31]				✓	✓		✓	✓	
van der Meij et al [32]				✓		✓			
Mundi et al [33]				✓				✓	
This study		✓	✓	✓	✓	✓	✓	✓	✓

a Element analyzed in the corresponding study.

Our remote follow-up system was developed based on relevant literature to maximize adherence and achieved an overall mean adherence rate of 87.37% (SD 15.37%). Comparison of this rate with previous studies is difficult, as most of these focused on adherence to a treatment or intervention rather than the use of an app. For example, Gomis-Pastor et al [34] showed that the use of an app significantly improved adherence to treatment and medication regimen, which led to significantly better symptom control, as found in a 2022 review [35]. However, Wikström et al [9] have shown that adherence to app use decreases over time, potentially due to improvement in health status or loss of interest [29], among other factors [26,29,32]. Our study aligns with these findings and shows a decrease in adherence from 94.53% in the pretreatment phase to 77.36% in the testing phase and 65.58% in the implantation phase [36-38].

### Limitations

The main limitation of our study is the specificity of the target population, as spinal cord stimulation is a specialized treatment for patients experiencing chronic pain. These patients often display a significant emotional component that can influence the outcomes of the interventions, posing challenges during designing this study and its remote follow-up system. In addition, these systems rely on new technologies that can be challenging for older patients, potentially limiting their ability to fully benefit from them. By using self-reported data, we also acknowledged that omissions or misunderstandings in the surveys may be introduced, which we attempted to minimize using validated scales. Finally, setting up a system similar to this study requires a significant financial investment.

### Conclusions

Digital systems are a part of medical care, and it is important that health care professionals are involved in the development of tools to ensure the achievement of desired standards. We have developed a remote monitoring system for patients undergoing spinal cord stimulation, based on scientific evidence and supported by a remote support center that improves treatment adherence. This system also provides us with detailed information about each patient. However, there is a tendency for patients to abandon the app usage over time, which could be related to long-term pain control. Further studies are needed to investigate the relationship between adherence and pain control.

## Abbreviations

DN4: Doleur Neuropathique 4
ODI/NDI: Oswestry Disability Index/Neck Disability Index
SF-36: 36-item Short Form Survey
VNRS: verbal numeral rating score
